# Kinetic studies on optimized extracellular laccase from *Trichoderma harzianum* PP389612 and its capabilities for azo dye removal

**DOI:** 10.1186/s12934-024-02412-2

**Published:** 2024-05-24

**Authors:** Amira Saad Abd El-latif, Abdel-Naser A. Zohri, Hamdy M. El-Aref, Ghada Abd-Elmonsef Mahmoud

**Affiliations:** 1 Molecular Biology Research and Studies Institute, Assiut University, Assiut, Egypt; 2 Botany and Microbiology Department, Faculty of Science, Assiut University, P.O. 71516, Assiut, Egypt; 3 Genetics Department, Faculty of Agriculture, Assiut University, Assiut, Egypt

**Keywords:** Laccase, *Trichoderma*, Statistical optimization, Bioremediation, Azo dyes

## Abstract

**Background:**

Azo dyes represent a common textile dye preferred for its high stability on fabrics in various harsh conditions. Although these dyes pose high-risk levels for all biological forms, fungal laccase is known as a green catalyst for its ability to oxidize numerous dyes.

**Methods:**

*Trichoderma* isolates were identified and tested for laccase production. Laccase production was optimized using Plackett–Burman Design. Laccase molecular weight and the kinetic properties of the enzyme, including K_m_ and V_max,_ pH, temperature, and ionic strength, were detected. Azo dye removal efficiency by laccase enzyme was detected for Congo red, methylene blue, and methyl orange.

**Results:**

Eight out of nine *Trichoderma* isolates were laccase producers. Laccase production efficiency was optimized by the superior strain *T. harzianum* PP389612, increasing production from 1.6 to 2.89 U/ml. In SDS-PAGE, purified laccases appear as a single protein band with a molecular weight of 41.00 kDa. K_m_ and V_max_ values were 146.12 μmol guaiacol and 3.82 μmol guaiacol/min. Its activity was stable in the pH range of 5–7, with an optimum temperature range of 40 to 50 °C, optimum ionic strength of 50 mM NaCl, and thermostability properties up to 90 °C. The decolorization efficiency of laccase was increased by increasing the time and reached its maximum after 72 h. The highest efficiency was achieved in Congo red decolorization, which reached 99% after 72 h, followed by methylene blue at 72%, while methyl orange decolorization efficiency was 68.5%.

**Conclusion:**

*Trichoderma* laccase can be used as an effective natural bio-agent for dye removal because it is stable and removes colors very well.

## Introduction

The laccase enzyme feature (EC 1.10.3.2) belongs to multicopper polyphenol oxidase; substrate specificity increases through a redox mediator’s addition to the reaction mixture [1, 2]**.** It works as a catalyst because of the copper atoms in the enzyme's structure: blue paramagnetic copper, non-blue paramagnetic copper, and two spin-coupled copper pairs [3]**.** Its stability is remarkable due to the carbohydrate structure portion, which protects the enzyme from inactivation by free radicals and proteolysis [4]**.** Laccase is a green catalyst because it oxidizes a wide range of xenobiotics and generates non-toxic water as a by-product [5]**.** It can oxidize synthetic dyes, azo dyes, phenolic compounds, herbicides, chlorinated phenols, dioxins, pesticides, cresols, and several pharmaceutical products [6]**.** Laccases are also used in the industry category as bioleaching for pulp, textile, and paper industries, biotransformation, and fruit juice clarification [7, 8]**.** Fungal laccases have a higher redox potential (E◦) than bacterial and plant laccases. This higher redox potential means that fungal laccases are more effective at bioremediation [9, 10]**.** Foreign substances and pollutants with a redox potential higher than 400 mV, like plastics, hydrocarbons (especially polycyclic), phenolic compounds, dyes, and other pharmaceutical compounds, can be broken down and taken out of the body by fungal laccase [11–13].

*Trichoderma* genus (*Hypocrea* in teleomorph) is thought to be a beneficial soil-borne fungus because it can improve both healthy and unhealthy soil quality, help plants grow, and work as a bioremediation and biocontrol agent against plant pathogens. It is also recommended as a safe, eco-friendly, and efficient enzyme producer that could be utilized as a promising choice for industrial and sustainable agriculture [14]. *Trichoderma* spp. develop rapidly on assorted substrates and generate massive green conidia, making them effectively identifiable and spreadable [15]. Some types of *Trichoderma*, like *T. harzianum* and *T. atroviride*, can make active laccases that can break down azo dyes and act as green catalysts [16, 17]. *Pleurotus ostreatus, Trametes versicolor,* and *Ganoderma lucidum* laccases are utilized for azo dye (Congo red) removal [9, 18, 19]. When *Fusarium oxysporum* laccase was present at a concentration of 100 mg/L, it was capable of eliminating 90% of malachite green [20].

These days, environmental contamination is speeding up daily due to fast urbanization and industrialization, generating unfavorable impacts on the natural environment [21–23]**.** Microplastics, organic dyes, pesticides, heavy metals, and hydrocarbons are all major environmental contaminants that cause severe effects on all living forms [24]**.** Amidst the previous contaminants, dyes occupied high priority as they are involved in textile industries, which contribute up to 15% of wastewater [25]**,** and it was reported that about 80% of textile wastewater containing dyes enters our water stream sources [26]. Dyes discharged on the waterbodies have an inconvenient environmental impact; creatures and people also exhibit signs of cell cytotoxicity, genotoxicity, and environmental ecotoxicity [27].

Azo dye N=N chromophore azo group gives its color properties to fabrics, and it is classified into mono, di, and poly according to the number of azo groups. Azo dyes represent common textile dyes that are preferable for their high stability on fabrics in various harsh conditions, and this explains their utilization in more than 70% of the textile industries [28]**.** In the meantime, its stability is a severe environmental issue threatening living organisms and the environment. The discharge of this dye type into the environment leads to carcinogenic and mutagenic effects on living tissues, including skin rash, allergies, jaundice, tumors, and heart defects [29]**.** Also, decreasing the photosynthetic activity and oxygen levels in aquatic plankton, zooplankton, phytoplankton, fish, and all aquatic life [21, 30].

Different chemical and physical strategies have been misused for dye and other contaminants elimination, like adsorption, constructed wetlands, cavitation, membrane filtration, ion exchange, ultrasound technique, oxidation, photocatalytic ozonation, precipitation, coagulation, flocculation, and electrochemical strategies [8, 31, 32]. Despite all these previous methods for dye removal, the removal process has a high cost and a high probability of secondary contamination; some require high energy and non-specificity, could be highly toxic, and some generate sludge problems [25, 33].

Bioremediation is still the most suitable, eco-friendly, low-cost, and effective solution for removing dyes. Microbes like bacteria, algae, fungi, and their metabolic enzymes are used in this process to get rid of contaminants by building up inside cells, changing them, bio-adsorbing them, or breaking them down [34, 35]. Using enzymes in bioremediation has recently received extraordinary attention in the academic and industrial sectors. In bioremediation, enzymes like oxidoreductases, hydrolases, oxygenases, peroxidases, halogenases, and esterases are very important. These enzymes are made by different microorganisms, mostly fungi [36]. The bio-degradation of azo dyes proceeds via extracellular or intracellular enzymatic cleavage, mainly laccase enzymes, which proved high efficiency in azo dye removal to less toxic compounds under different environmental conditions, like cleaving azo dye to form non-toxic nitrogen compounds [28].

*Trichoderma* species have a high ability to produce active laccase, known as eco-friendly green catalyst. Although fungal laccase has high efficiency in azo dye removal, only a few research studies have discussed its properties in dye removal, especially from *Trichoderma*. The current study investigates laccase production from different *Trichoderma* species and optimises the production process for high efficiency using Plackett–Burman Design (PBD). Optimized *Trichoderma* laccase was extracted and characterized, and some kinetic properties were investigated. The last part concerns the application of fungal laccase as azo dye removal for three different azo dyes.

## Materials and methods

### *Trichoderma* collection and identification

Nine isolates of *Trichoderma* species were isolated from mushroom waste, tomato rhizoplane, onion and wheat rhizosphere, heavy metal-polluted soil, and agriculture soil in Assiut Governorate, Egypt. The plants were uprooted and shaken for rhizosphere samples to collect the soil-adhering roots, while the rhizoplane samples were uprooted and the roots were collected. After taking the samples, they were put in clean polyethylene bags and transferred to the laboratory for fungal isolation [37]. *Trichoderma* was isolated on Czapek’s dextrose agar medium (3% sucrose, 0.2% NaNO_3_, 0.1% KCl, 0.05% MgSO_4_.7H_2_O, 0.001% FeSO_4_, 0.001% ZnSO_4_, 0.0005% CuSO_4_, and 2% agar) using direct and dilution plate methods. In brief, for the direct method (used for mushroom waste and tomato rhizoplane), the plant samples were cut into approximately one cm^2^ equal segments and subjected to washing with 5% sodium hypochlorite, then washed with sterilized distilled water, dried between sterilized filter papers, and placed on the surface of Czapek’s dextrose agar medium [38]. For rhizosphere and soil samples, the dilution plate method was performed. One gram of root-adhering soil was mixed with 9 ml of sterilized distilled water and shaken for 10 min. Then serial dilutions were made, and one ml of the proper dilution was transferred to a sterilized Petri dish and covered with a medium. All plates were incubated at 28 ± 1 °C for one week, and the developed green colonies were examined for selecting *Trichoderma* species [38–40]. Developed *Trichoderma* were selected, re-cultured in new plates, and identified through their macroscopic (colonies color, size, and reverse) and microscopic features (conidiophores, branching, phialides, and conidia) following the identification keys of Bissett [41], Harman, and Kubicek [42].

### Qualitative determination of laccase produced by *Trichoderma* isolates

*Trichoderma* isolates were primarily screened qualitatively to detect their capabilities for laccase production using Czapek’s dextrose agar medium supplemented with 0.04% guaiacol and 150 μg/l antibacterial agents (chloramphenicol) separately sterilized using membrane filtration (pore size 0.22 mm). The medium was prepared and then sterilized in an autoclave at 121 °C for 15 min. After cooling to approximately 45–50 °C, it was poured into sterile petri plates, inoculated with three-day-old *Trichoderma* species and incubated at 28 °C for 6 days in a static incubator. The ability to produce laccase will be visualized as reddish-brown zones in the agar medium [43].

### Quantitative determination of laccase produced by *Trichoderma* isolates

*Trichoderma* isolates were cultivated in a modified guaiacol mineral medium containing 0.3% peptone, 1% glucose, 0.06% KH_2_PO_4_, 0.04% K_2_HPO_4_, 0.05% MgSO_4_, 0.02% guaiacol, and trace elements (0.05 MnSO_4_, 0.001 ZnSO_4_, and 0.0005 FeSO_4_ g/l) [44]. The medium was prepared and then sterilized in an autoclave at 121 °C for 15 min. After cooling, the mineral sterilized medium was inoculated by 2% three-day-old *Trichoderma* isolates (10^6^ spores/ml) and kept at 28 °C for 6 days in a 200-rpm rotary incubator. After 6 days, the biomasses were removed, the supernatants were centrifuged at a cooling temperature of 4 °C with 6000 × g for 10 min, and clear supernatants were set for laccase activity determination.

### Laccase activity assay

The prepared *Trichoderma* supernatants were used to measure extracellular laccase (U/ml) following [43, 45] the method of substrate oxidation (guaiacol) with some modifications. There are 3 ml of 50 mM sodium acetate buffer (pH 4.5), 100 μl of guaiacol, and 1.9 mL of extracellular enzyme supernatant in the reaction tubes. The tubes were homogenized and kept at 40 °C for 5 min. The developed reddish-brown color absorbance was measured using a spectrophotometer at 465 nm. The formula calculated laccase activity (U/ml):

1 $${\text{Laccase activity }}\left( {{\text{U}}/{\text{ml}}} \right) = \left( {{\text{A}} * {\text{V1}}} \right)/ \left( {{\text{t }} * \EUR * {\text{V2}}} \right)$$

where A is absorbance, V1 is the reaction volume (ml), t is the incubation time (5 min), € is the extinction coefficient at 465 (12,100 M − 1 cm − 1), and V2 is the enzyme volume (1.9 mL). One unit of laccase defined as the amount of enzyme that required to oxidize 1 µmole of guaiacol per minute at a temperature of 40 °C.

### Molecular identification of *Trichoderma harizanum* (PP389612)

*Trichoderma harizanum* (PP389612) was re-cultivated on Czapek’s dextrose agar plates and kept at 25 °C for 3 days. Mycelia were then harvested and used for genomic DNA extraction according to previously described methods [46]. 50 mg of *T. harizanum* mycelium was placed into 1500 μl tubes with glass beads, followed by 200 μl of lysis buffer and 20 μl of Proteinase K, and kept at 56 °C for 180 min. Then, tubes were fortified with 100 μl of a 3.0 M potassium acetate solution (pH 6.5) and centrifuged for 2 min. After transferring 400 μl of the supernatant to a fresh tube containing 500 μl isopropanol and centrifuging it for two minutes, the DNA pellets were cleaned, rinsed with 750 μl ethanol, then centrifuged for one minute to precipitate the DNA. After the DNA pellet was air-dried, the isolated DNA was stored at − 70 °C and analyzed through SolGent (Daejeon, South Korea) for PCR amplification and DNA sequencing.

The ITS (internal transcribed spacer) region of rDNA was amplified via two types of universal primers: ITS1 (5'—TCC GTA GGT GAA CCT GCG G—3') and ITS4 (5'—TCC TCC GCT TAT TGA TAT GC—3'). The mixtures were prepared using SolGent EF-Taq as follows: 10 mM dNTP (T) 0.5 µl, 10X EF-Taq buffer 2.5 µl, primer (F-10p) 1.0 µl, primer (R-10p) 1.0 µl, DNA template 1.0 µl, DW to 25 µl. Amplification was performed on ABI 9700 thermal cyclers. Programmed for an initial denaturation at 95 °C for 15 min, 30 cycles of 20-s denaturation at 95 °C, 40-s annealing at 50 °C, and 1 min extension at 72 °C, followed by a final extension for 5 min at 72 °C. The products of PCR purification were confirmed by 1% agarose gel electrophoresis using a size marker. Samples were sequenced using the same primers for sense and antisense directions, and contigs were obtained through CLCBio Main Workbench software. The generated sequences were analyzed through the National Center for Biotechnology Information’s (NCBI) BLAST website, and the isolate's accession numbers were set in the GenBank database. Also, the sequences were analyzed through Clustal W by MegAlign software, 5.05, for the phylogenetic tree drawing [47].

### Enhancing laccase production condition using Plackett–Burman design (PBD)

*Trichoderma harzianum* PP389612, recorded as the highest laccase producer in this study and recovered from heavy metal-polluted soil, was chosen for the enhancement experiment. Plackett–Burman Design (PBD) was set to optimize the medium components and the growth conditions for high laccase production and evaluate the tested parameter interactions and their main effects. Eleven parameters with low (−1) and high levels (+ 1), 13 runs, and one central point were established. The growth condition and nutrition independent variables of laccase were A: Incubation temperature (°C), B: Incubation time (d.), C: Fermentation type (shaking or static), D: Inoculum size (%), E: Initial pH, F: Fermentation volume (ml), G: Glucose (g/l), H: Peptone (g/l), J: KH_2_PO_4_ (g/l), K: K_2_HPO_4_ (g/l), and L: Guaiacol (μg/l), as shown in Table 1. Each conical flask (250 ml) contains the specific medium constituents and is incubated at the specific conditions following the design and inoculated with *T. harzianum* spores (10^6^ spores/ml). The design utilized for multifactor and quick selection for the highest affects independent factors using the first-order polynomial equation [48, 49]:

2 $${\text{Y }} = \, \beta 0 \, + \, \sum \, \beta {\text{i xi}}$$

**Table 1:** Plackett–Burman design for screening the most significant parameters affecting on the laccase production by *Trichoderma harzianum* (PP389612)

Run	A Incubation temperature (°C )	B Incubation time (d.)	C Fermentation type	D Inoculum size (%)	E Initial pH	F Fermentation Volume	G Glucose (g/l)	H Peptone (g/l)	J KH_2_PO_4_ (g/l)	K K_2_HPO_4_ (g/l)	L Guaicol (μg/l)	Laccase enzyme (U/ml) Predicted	Laccase enzyme (U/ml) Actual
1	25	8	Static	2	5	50	5	6	0.1	1	300	1.64	1.54
2	35	4	Shaking	0.5	7	50	15	6	0.1	1	300	1.4	1.37
3	30	6	Shaking	1	6	100	10	3	0.5	0.5	100	1.99	1.69
4	25	8	Static	0.5	7	200	15	1	0.1	0.1	300	1.33	1.45
5	35	8	Static	0.5	5	50	15	1	1	1	50	2.61	2.75
6	25	8	Shaking	2	7	50	15	6	1	0.1	50	2.71	2.89
7	35	4	Static	2	5	200	15	6	0.1	0.1	50	2.42	2.37
8	25	4	Static	0.5	7	200	5	6	1	1	50	1.63	1.73
9	35	8	Shaking	0.5	5	200	5	6	1	0.1	300	2.75	2.81
10	25	4	Shaking	2	5	200	15	1	1	1	300	0.77	0.77
11	35	8	Shaking	2	7	200	5	1	0.1	1	50	2.18	2.05
12	35	4	Static	2	7	50	5	1	1	0.1	300	2.31	2.24
13	25	4	Shaking	0.5	5	50	5	1	0.1	0.1	50	1.74	1.81

Y represents the predicted laccase enzyme response, β0 and βi represent constant coefficients for design, and xi is the coded independent multifactor. 3D surfaces and standard plots will be set to clear the relationships between the eleven variables.

### SDS-PAGE analysis for laccase enzyme

The enzyme was precipitated following Assavanig et al. [50] using cold ethanol (99.0%) with a triple volume ratio and preserved at 4 ºC. The precipitate was then suspended in 50 mM citrate buffer at pH 7.0 and rewashed with 70% ethanol and freeze-dried. A 0.5 g enzyme powder was dissolved in 5 mL of 50 mM sodium acetate buffer (pH 4.5) and dialyzed (cutoffs: 12–14 KD) against deionized water [51] and utilized as pure fungal laccase in characterization and application tests. Polyacrylamide (PAGE) gel electrophoresis was performed to assess laccase’s purity and molecular weight. SDS-PAGE was carried out following the protocol of Laemmli [52], resolving gel (12%) and stacking gel (5%) using a vertical gel electrophoresis system containing SDS (0.1%). For enzyme preparation, 0.1 g of laccase (purified laccase) dissolved in 1 mL buffer solution (0.5 M tris–HCl) was vortexed for 30 s, then 10 μL was taken from the solution and mixed with 10 μl of SDS (0.5%) and beta-mercaptoethanol (5%), this mixture was boiled at 100 °C for 5 min before loading onto the gel. Protein bands were visualized by staining the gel with Coomassie Brilliant Blue R250 stain (one gram of stain dissolved in methanol-acetic acid–water solution (4:1:5, v:v). The relative molecular weight was assessed by calculating the relative mobility of protein markers to the sample in the run.

### Kinetics studies on laccase

#### Michaelis constant (K_m_) and maximum velocity (V_max_) determination

For laccase Michaels constant determination, different guaiacol concentrations were used individually in a reaction mixture with 10, 50, 100, 200, 300, 400, 500, 600, 700, 800, 900, and 1000 μl concentrations. The rest of the reaction mixture at each guaiacol concentration involves 3 ml of 50 mM sodium acetate buffer with pH 4.5 and 1.9 mL of extracellular enzyme. The reaction was set for 5 min at 40 °C, and laccase activity was determined using a spectrophotometer as previously. K_m_ and V_max_ values of laccase were calculated via the Lineweaver-Bulk curve of guaiacol concentrations against laccase activity [53–55].

#### pH, temperature, and ionic strength tolerance of laccase

The enzyme pH-dependency of the activity was detected in acidic and alkaline ranges at 3, 4, 5, 7, and 9 pH. The enzyme activity was tested for its thermostability at 10, 20, 30, 40, 50, 60, 70, 80, and 90 °C. The ionic strength test was conducted using different NaCl concentrations: 0, 100, 200, 300, 400, 500, 600, 700, 800, 900, and 1000 mM [55].

### Azo dyes degradation assay

Three azo dyes were utilized in this test: Congo red, methylene blue, and methyl orange [28]. The dyes were tested at 50 mg/L concentrations (5 mg dye was dissolved in 100 ml sodium acetate buffer, PH 4.5) and 20 mg purified laccase (1.922 U/mL) at 30 °C. The absorbance of azo dyes was taken using an ultraviolet spectrophotometer according to the time interval of 3 days at 485 nm for Congo red, 618 nm for methylene blue, and 485 nm for methyl orange. Also, different azo-dyes concentrations (5–300 mg/L) were tested for decolorization efficiency after 72 h. The decolorization percentage is calculated following the formula:

D (%) = (Ai − At)/(Ai × 100), where D-degradation, Ai-initial absorbance, and At-Absorbance after time t.

### Statistical analysis

All the optimization data were statistically analyzed through multiple regression statistical analysis by Design Expert 7.0.0 software, United States. A one-way ANOVA is also set with a 0.05 probability to illustrate the data regression and variable interactions.

## Results

### Phenotypic characteristics of *Trichoderma* species

**Fig. 1:**
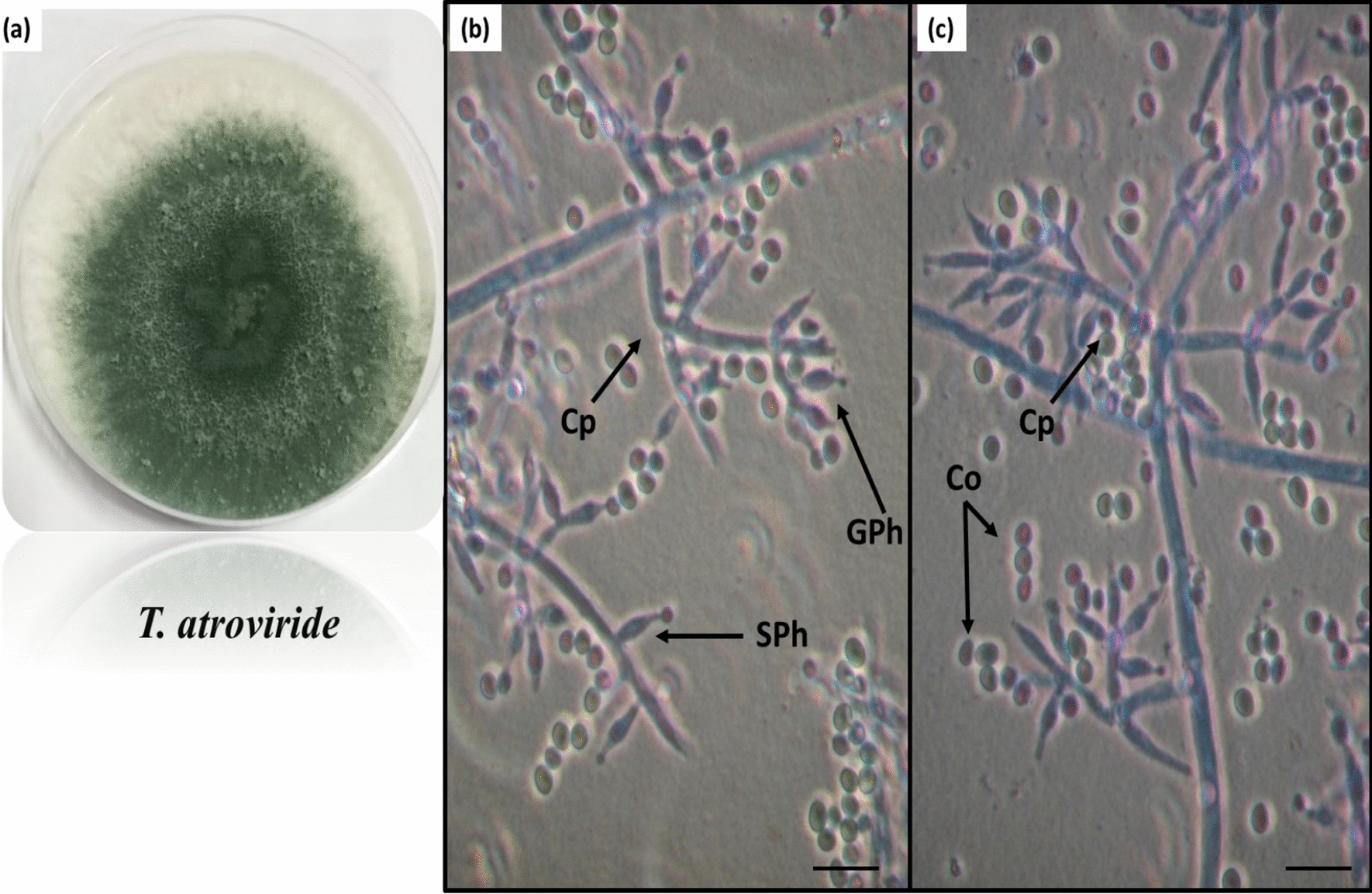
*Trichoderma atroviride* P. Karsten, growth on Czapek’s dextrose agar medium showing the aerial mycelia (**a**), branched conidiophores (Cp), solitary phialides (SPh), grouped phialides (GPh), and sub-globose conidia (Co) cleared in **b**, **c** figures, scale bar, 10 μm

**Fig. 2:**
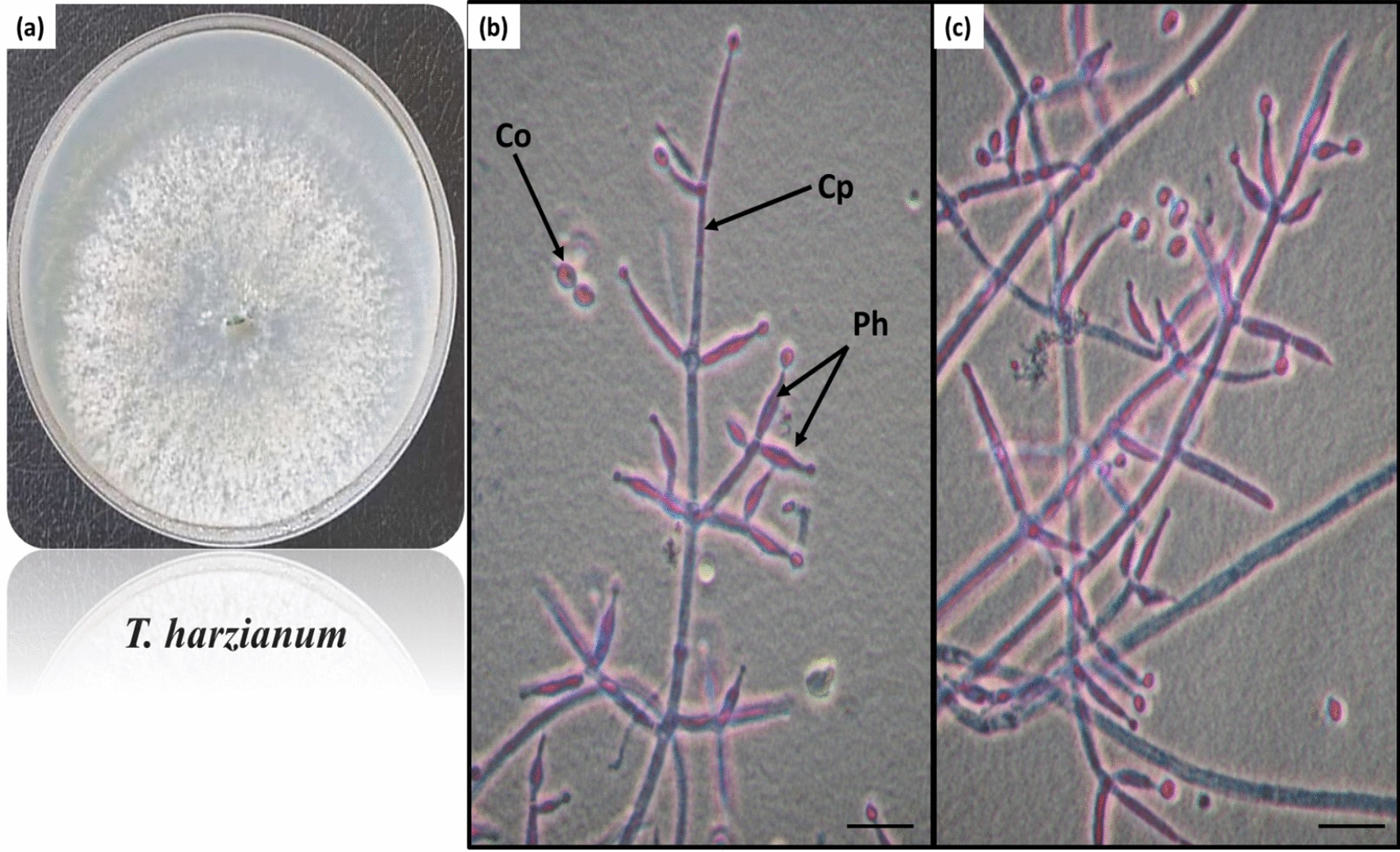
*Trichoderma harzianum* Rifai, growth on Czapek’s dextrose agar medium showing aerial mycelia (**a**), highly branched giving loose tufts shapes conidiophores (Cp), singl phialides (Ph), and sub-globose to ovoid conidia (Co) cleared in **b**, **c** figures, scale bar, 10 μm

**Fig. 3:**
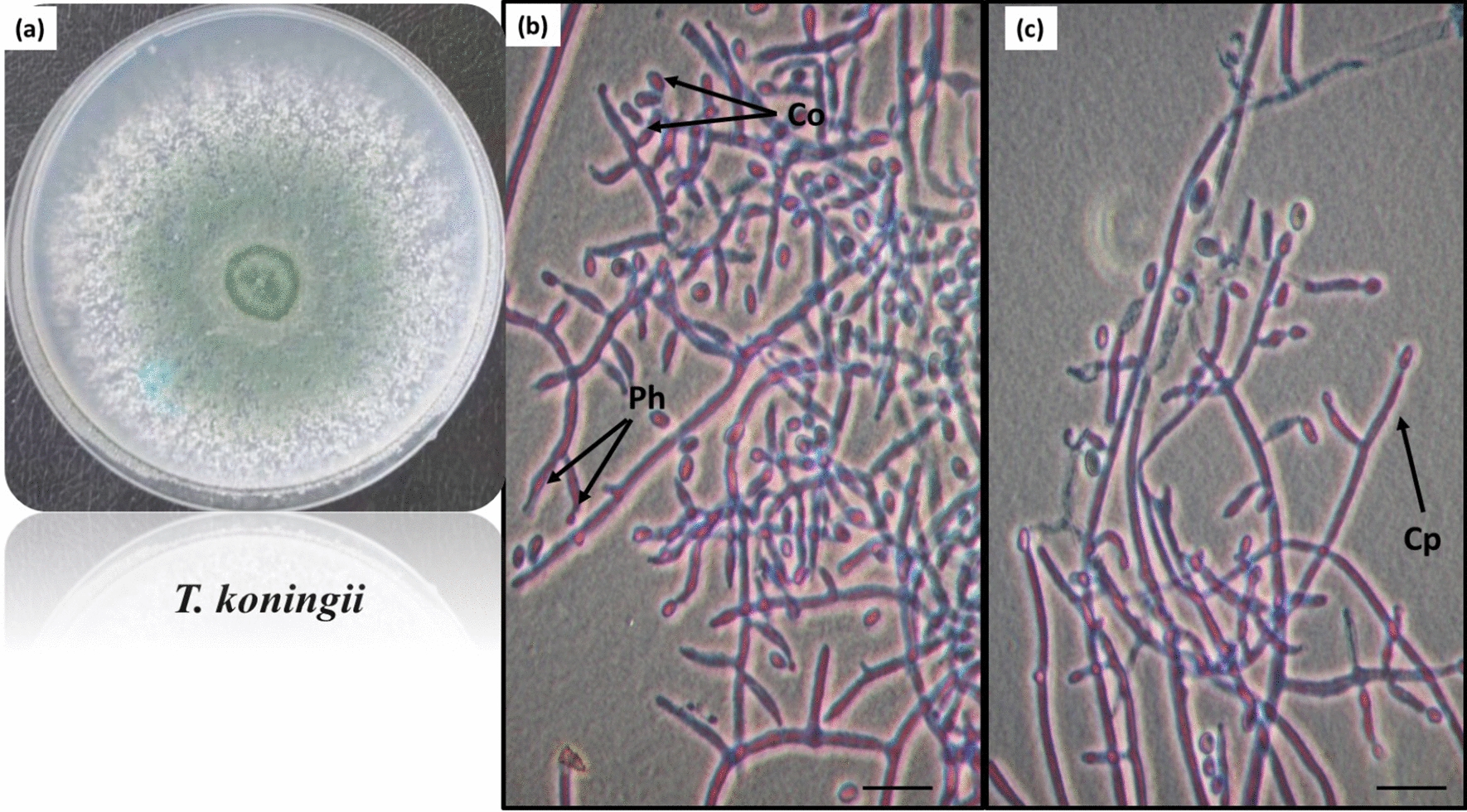
*Trichoderma koningii* Oud., growth on Czapek’s dextrose agar medium showing aerial mycelia (**a**), broader and more rigid conidiophores (Cp), lageniform or ampulliform phialides (Ph), and subcylindrical to ellipsoid conidia (Co) cleared in **b**, **c** figures, scale bar, 10 μm

The nine isolates of *Trichoderma* were identified as phenotypic using macroscopic and microscopic features. The isolates came from three species: T. *atroviride* (ASU 112, ASU 423, ASU 333, ASU 216), T. *harzianum* (AUMC 16189, AUMC 16206, PP389612, ASU 314), and T. *koningii* (ASU 118). Their shapes are shown in Figs. 1, 2 and 3.

- *1. Trichoderma atroviride* P. Karsten, morphological description.

Granular surface mycelial growth rises on Czapek’s dextrose agar medium and changes rapidly from white to dark green with a 5–8 cm diameter after 5 days. The reverse is uncolored and may turn dull yellowish with age. The conidiophores (Cp) have branches, and the phialides (Ph) can be solitary, grouped up to four verticillates, and often curved. They are 6–11 × 2.5–3.0 μm in size. Conidia (Co) are dark green with smooth walls and sub-globoses ranging from 2.7–3.8 × 2.1–3.5 μm (Fig. 1).

- *2. Trichoderma harzianum* Rifai, morphological description.

Smooth mycelial growth rises on Czapek’s dextrose agar medium and changes from white to light green after two days, with a diameter of 8–9 cm after 5 days; there is no reverse production. Conidiophores (Cp) are highly branched, giving loose tuft shapes, and phialides (Ph) are singly rises with a short, skittle shape range of 7–11 × 2.5–3 μm. Conidia (Co) are sub-globose to ovoid with smooth walls and truncate bases ranging from 2.6 to 3.3 × 2.4 to 3 μm (Fig. 2).

- *3. Trichoderma koningii* Oud., morphological description.

Granular surface mycelial growth rises on Czapek’s dextrose agar medium with continuous crusts, a dull green to bluish-green color, and a 7–9 cm diameter. Reverse uncolored and could turn yellowish with aging. Conidiophores (Cp) have broader and more rigid elements that verticillate. Phialides (Ph) are usually grouped in two verticillate or 3–5-verticillate in some strains, lageniform or ampulliform, 7.4–12 × 2.5–3.4 μm. Conidia (Co) are green with smooth walls, subcylindrical to ellipsoid, ranging from 3–5.2 × 1.9–3.1 μm (Fig. 3).

**Fig. 4:**
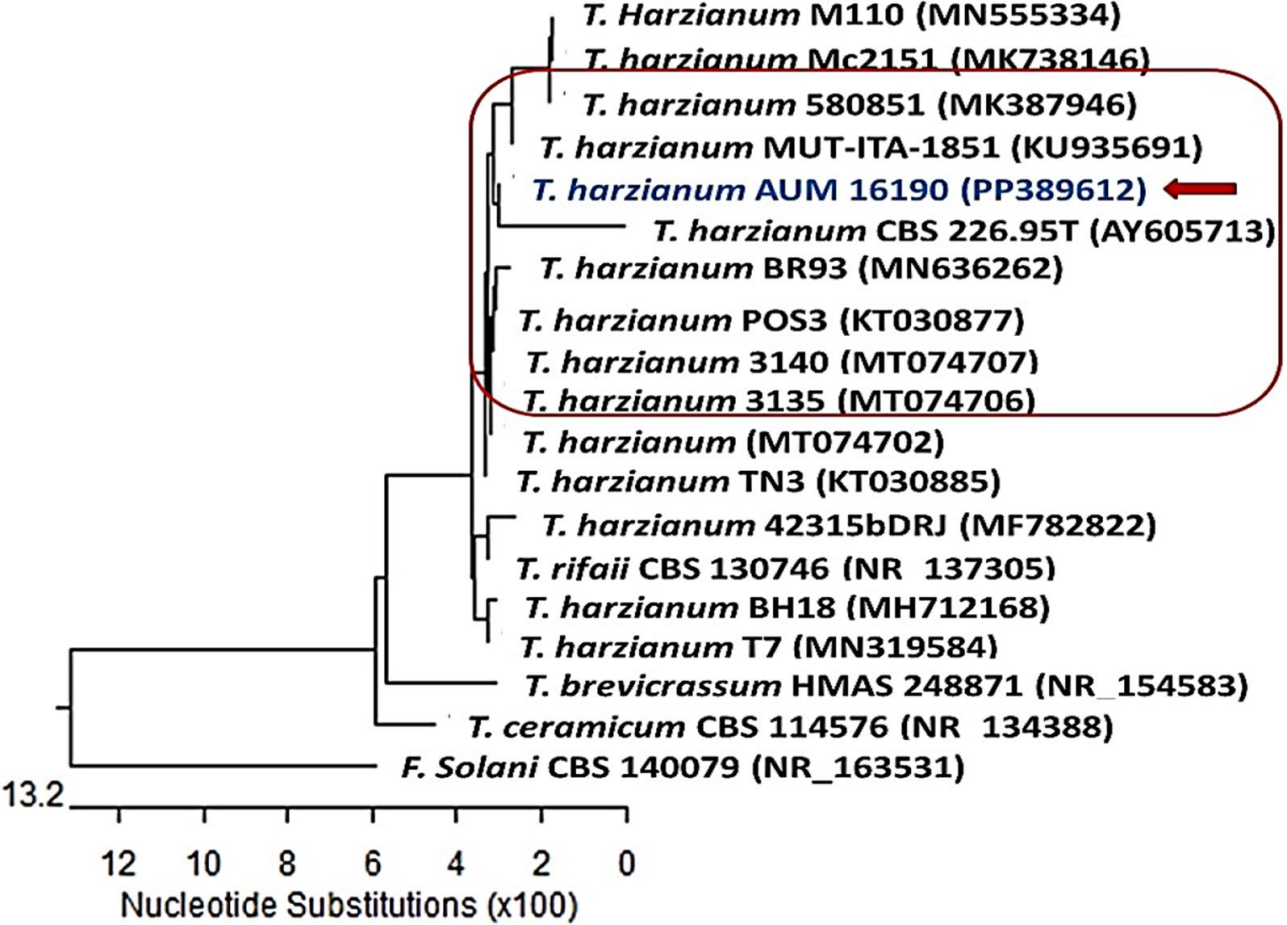
Phylogenetic tree based on ITS sequences of rDNA of the fungal sample isolated in the present study (*Trichoderma harzianum* PP389612) aligned with closely related strains accessed from the GenBank. This strain showed 99.83% identity and 100% coverage with several strains of the same species including the type material *T. harzianum* CBS 22695 with GenBank accession no. AY605713. *Fusarium solani* represents an outgroup strain. F. = *Fusarium, T.* = *Trichoderma*

The identification of *Trichoderma harzianum* (PP389612), the highest laccase producer, has been confirmed by genetic analysis. Molecular identification data showed that *Trichoderma harzianum* (PP389612) has 586 base pairs with 100% matching with *Trichoderma harzianum* [KU935691] and 99.8% matching with *T. harzianum* [MK738146], *T. harzianum* [MT074706], *T. harzianum* [MT074707], and *T. harzianum* [MN555334] as shown in the phylogenetic tree (Fig. 4) and the isolate identified as and sets in the gene bank with accession number [PP389612].

### Screening for extracellular laccase production by *Trichoderma* isolates

Nine *Trichoderma* isolates collected from heavy metal-polluted soil, mushroom waste, onion and wheat rhizospheres, tomato rhizoplanes and agriculture soil in Assiut Governorate, Egypt, were tested for laccase production. Eight of nine *Trichoderma* isolates showed laccase production, as shown in Table 2. The positive isolates were related to *T. harzianum* and *T. atroviride,* while *T. koningii* did not show laccase activity. *Trichoderma harzianum* (PP389612), which was isolated from heavy metal-polluted soil, was the highest laccase producer, giving 1.6 ± 0.045 U/ml, followed by *T. atroviride* (ASU 333) with 1.43 ± 0.079 U/ml. *Trichoderma harzianum* (ASU 314), *T. harzianum* (AUMC 16206), *T. atroviride* (ASU 423), and *T. atroviride* (ASU 216) give 1.24 ± 0.022, 1.23 ± 0.058, 1.22 ± 0.089, and 1.21 ± 0.13 U/ml activity, respectively. However, *T. harzianum* AUMC 16189 and *T. atroviride* ASU 112 give low production by 0.83 ± 0.01 and 0.55 ± 0.02 U/ml, respectively.

**Table 2:** Qualitative and quantitative detection of laccase enzyme by *Trichoderma* species in guaiacol agar and liquid media

No	Name	Isolate number	Laccase enzyme			
			Isolation source	Primary screening (mm)	Laccase activity (U/ml)	Dry mass (g/l)
1	*T. harzianum*	AUMC 16189	Onion rhizosphere	13 ± 1	0.83 ± 0.01	2.65 ± 0.04
2	*T. harzianum*	AUMC 16206	Agriculture Soil	50.5 ± 2.5	1.23 ± 0.058	3.56 ± 0.016
3	*T. harzianum*	PP389612	Heavy metal polluted soil	86 ± 1	1.6 ± 0.045	5.08 ± 0.15
4	*T. harzianum*	ASU 314	Mushroom wastes	64 ± 1	1.24 ± 0.022	3.56 ± 0.03
5	*T. atroviride*	ASU 112	Onion rhizosphere	21 ± 2	0.55 ± 0.02	1.48 ± 0.23
6	*T. atroviride*	ASU 423	Heavy metal polluted soil	73 ± 3	1.22 ± 0.089	3.46 ± 0.035
7	*T. atroviride*	ASU 333	Wheat rhizosphere	85.5 ± 3.5	1.43 ± 0.079	4.43 ± 0.32
8	*T. atroviride*	ASU 216	Tomato rhizoplane	46 ± 2	1.21 ± 0.13	3.77 ± 0.14
9	*T. koningii*	ASU 118	Onion rhizosphere	0.0	0.0	0.0

### Enhancing laccase production using experimental designs

The Plackett–Burman design was set to choose the highly significant independent factors using the first-order polynomial equation for extracellular laccase production by *Trichoderma harzianum* (PP389612). Eleven different variables were tested: incubation temperature (A, ゜C), Incubation time (B, d.), Fermentation type (C, shaking or static), Inoculum size (D, %), Initial pH (E), Fermentation volume (F, ml), Glucose (G, g/l), Peptone (H, g/l), KH_2_PO_4_ (J, g/l), K_2_HPO_4_ (K, g/l), and Guaicol (L, μg/l). For laccase production, the model used the following first-order polynomial:

3 $${\text{Laccase production }}\left( {{\text{U}}/{\text{ml}}} \right){\mkern 1mu} = {\mkern 1mu} 1.96 + {\mkern 1mu} \left( {0.32} \right){\mkern 1mu} * {\text{ A }} + {\mkern 1mu} \left( {0.24} \right){\mkern 1mu} * {\text{ B }} + {\mkern 1mu} \left( {0.03} \right){\mkern 1mu} * {\text{ C }} + {\mkern 1mu} \left( {0.047} \right){\mkern 1mu} * {\text{ D }} + {\mkern 1mu} \left( { - 0.031} \right){\mkern 1mu} * {\text{ E }} + \,\,{\mkern 1mu} \left( { - 0.11} \right){\mkern 1mu} * {\text{ F }} + {\mkern 1mu} \left( { - 0.084} \right){\mkern 1mu} * {\text{ G }} + {\mkern 1mu} \left( {0.134} \right){\mkern 1mu} * {\text{ H }} + {\mkern 1mu} \left( {0.172} \right){\mkern 1mu} * {\text{ J }} + {\mkern 1mu} \left( { - 0.252} \right){\mkern 1mu} * {\text{ K }} + {\mkern 1mu} \left( { - 0.257} \right){\mkern 1mu} * {\text{ L}}$$

**Fig. 5:**
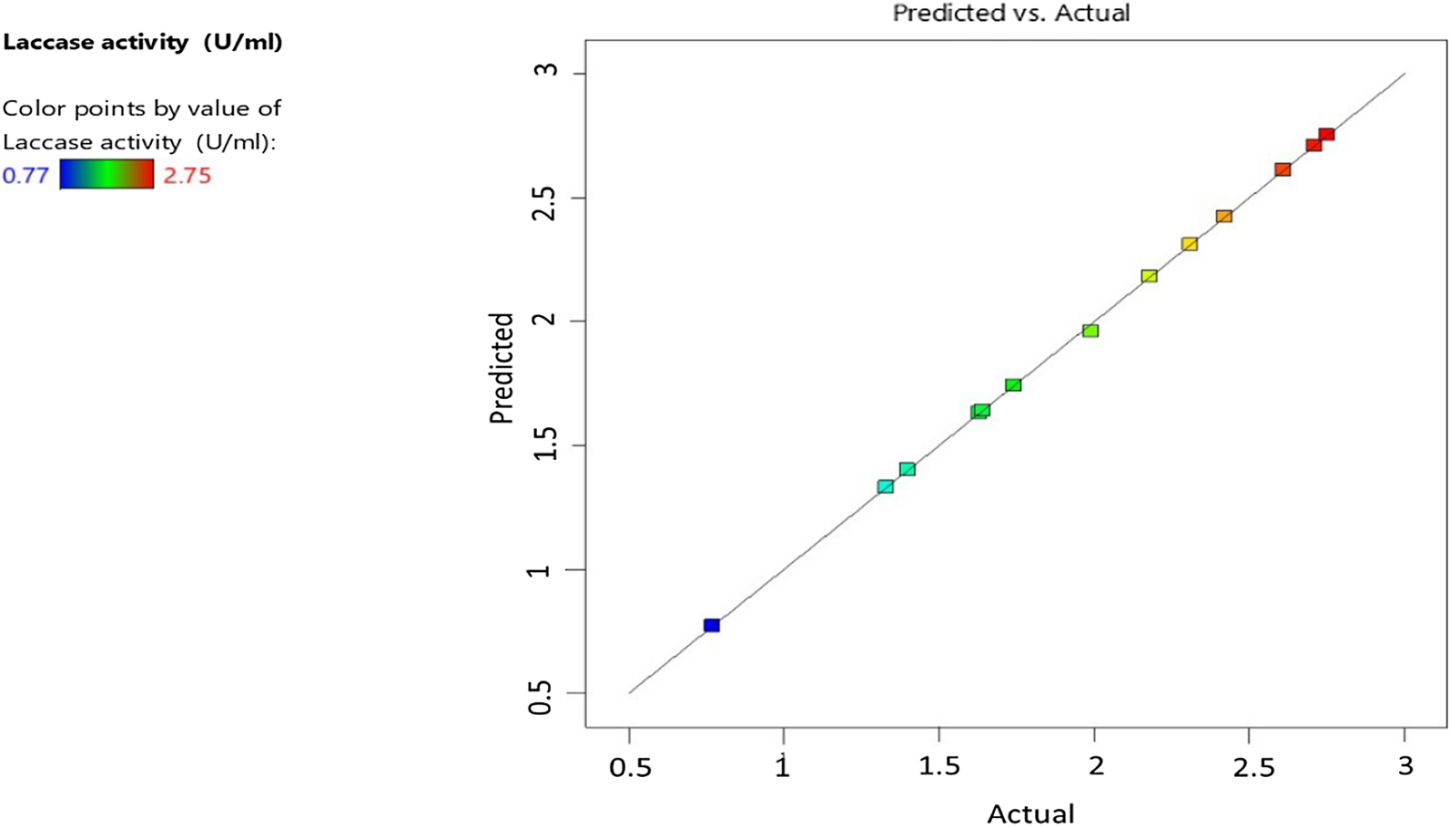
Predicted and the actual data comprised in Plackett–Burman design of the laccase production by *Trichoderma harzianum* (PP389612)

The significant variables that affect highly in the laccase production process (p < 0.05) were incubation temperature (A), incubation time (B), peptone (H), KH_2_PO_4_ (J), K_2_HPO_4_ (K), and guaiacol (L), In contrast, the highest significance was achieved by incubation temperature (A). However, fermentation type (C), inoculum size (D), initial pH (E), fermentation volume (F), and glucose (G) were non-significant (p > 0.05) for the production process, as presented in the ANOVA analysis in Table 3. The highest laccase production was 2.89 U/ml (predicted value: 2.71 U/ml) obtained in run (6) using incubation temperature (25 °C), incubation time (8 days), fermentation type (shaking), inoculum size (2%), initial pH (7), fermentation volume (50 ml), glucose (15 g/l), peptone (6 g/l), KH_2_PO_4_ (1 g/l), K_2_HPO_4_ (0.1 g/l), and guaiacol (50 μg/l). The predicted results of the Plackett–Burman design were close to the experimental data, as shown in Table 1 and Fig. 5, which collaboratively made the design adequate to explain the data significance and variations. The Plackett–Burman model *F* and *P*-values of laccase enzyme production were 408.19 and *P* < 0.05, In contrast, the coefficient (*R*^*2*^) was 0.999, and the adjusted (*R*^*2*^) was 0.997, indicating the high significance and adequateness of the design to explain the data effects and variations.

**Table 3:** ANOVA results for Plackett–Burman design of the laccase production by *Trichoderma harzianum* (PP389612)

Source	Sum of squares	df	Mean square	F-value	p-value
Model	4.38	11	0.3980	408.19	0.0386
A-incubation temperature (゜C)	1.24	1	1.24	1266.88	0.0179
B-incubation time (d.)	0.7252	1	0.7252	743.80	0.0233
C-fermentation type	0.0127	1	0.0127	13.00	0.1722
D-inoculum size (%)	0.0271	1	0.0271	27.77	0.1194
E-initial pH	0.0114	1	0.0114	11.70	0.1811
F-fermentation volume	0.1474	1	0.1474	151.19	0.0517
G-glucose (g/l)	0.0850	1	0.0850	87.19	0.0679
H-peptone (g/l)	0.2160	1	0.2160	221.55	0.0427
J-KH_2_PO_4_ (g/l)	0.3571	1	0.3571	366.23	0.0332
K-K_2_HPO_4_ (g/l)	0.7651	1	0.7651	784.69	0.0227
L-guaicol (μg/l)	0.7957	1	0.7957	816.08	0.0223

**Fig. 6:**
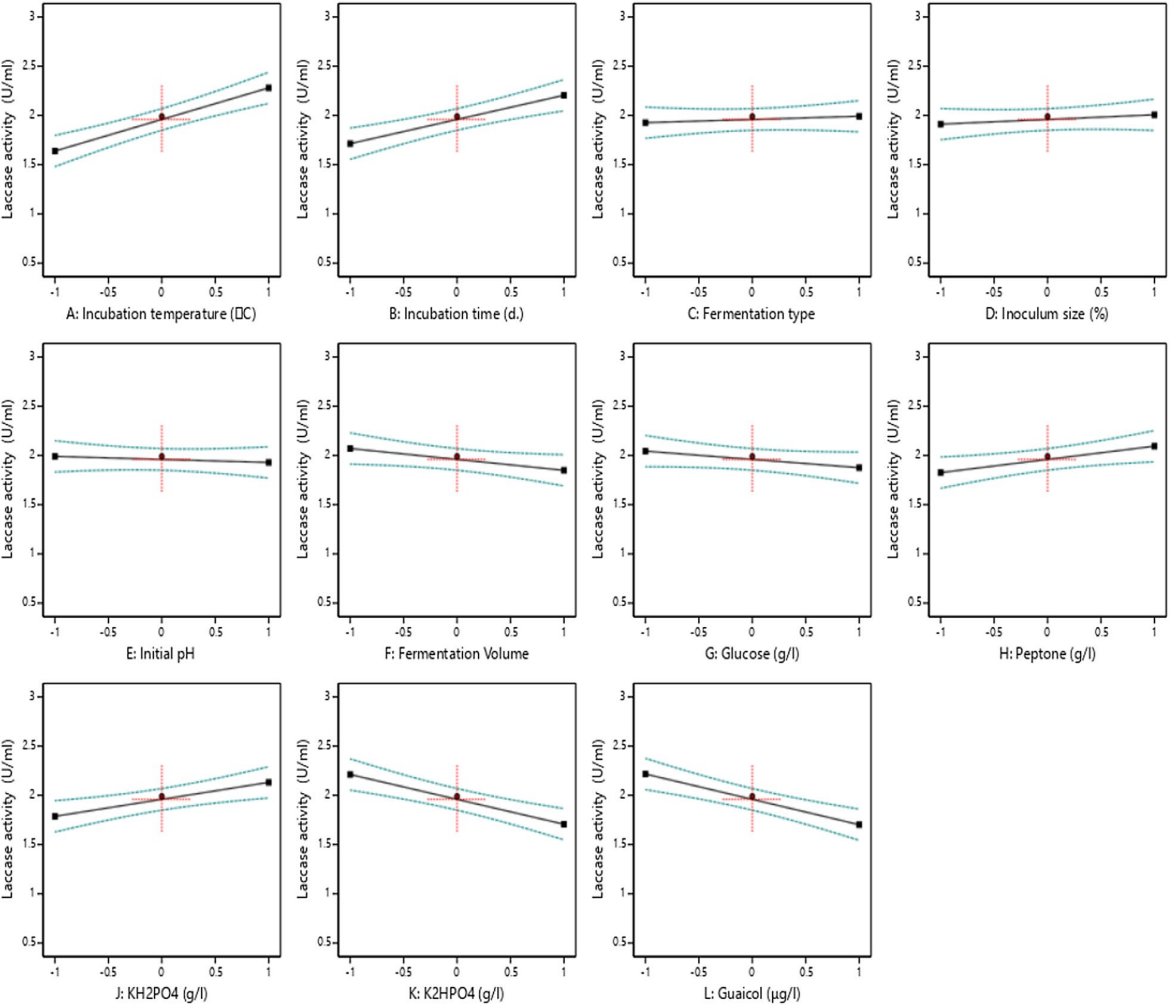
The main effects in Plackett–Burman design of each variable from the eleven variables on laccase production by *Trichoderma harzianum* (PP389612)

**Fig. 7:**
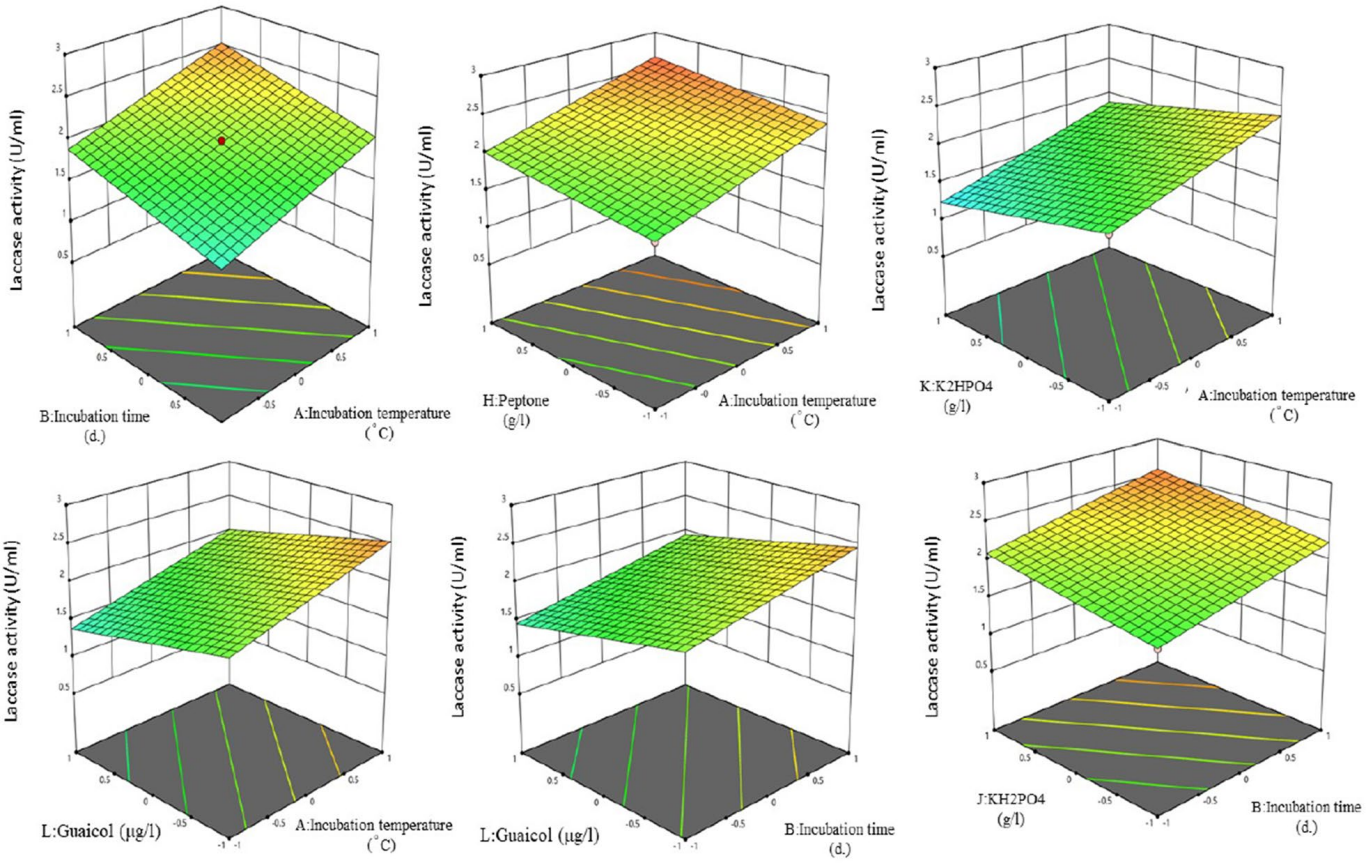
Plackett–Burman design 3D plots of the interaction effects between two variables where other variables remain at zero coded for laccase production by *Trichoderma harzianum* (PP389612). The figures clear the interactions between AB (Incubation temperature * Incubation time), AH (Incubation temperature * Peptone), AK (Incubation temperature * K_2_HPO_4_), AL (Incubation temperature * Guaicol), BL (Incubation time* Guaicol), and BJ (Incubation time* KH_2_PO_4_)

The main effects of each variable on laccase production by *Trichoderma harzianum* (PP389612) are shown in Fig. 6. The enzyme activity increases by increasing these individual parameters: incubation temperature (**A**), incubation time (B), peptone (H), and KH_2_PO_4_ (J, g/l), while increasing by decreasing fermentation volume (F), glucose (G), K_2_HPO_4_ (K), and guaiacol (L). However, fermentation type (C), Inoculum size (D), and Initial pH (E) did not give clear directions. Plackett–Burman designed 3D plots of the interaction effects between two variables where other variables remain at zero coded for laccase production by *Trichoderma harzianum* (PP389612), as shown in Fig. 7. The interaction takes a positive direction in the cases of AB (Incubation temperature * Incubation time), AH (Incubation temperature * Peptone), and BJ (Incubation time* KH_2_PO_4_), where the laccase activity increases by increasing the values of both parameters. However, in AL (Incubation temperature * Guaiacol) and BL (Incubation time* Guaiacol), by increasing the temperature and incubation time and decreasing the substrate concentration, the enzyme activity increased, while increasing the temperature and decreasing K_2_HPO_4_ in AK (Incubation temperature * K_2_HPO_4_) interaction increased the enzyme activity.

### SDS-PAGE analysis

**Fig. 8:**
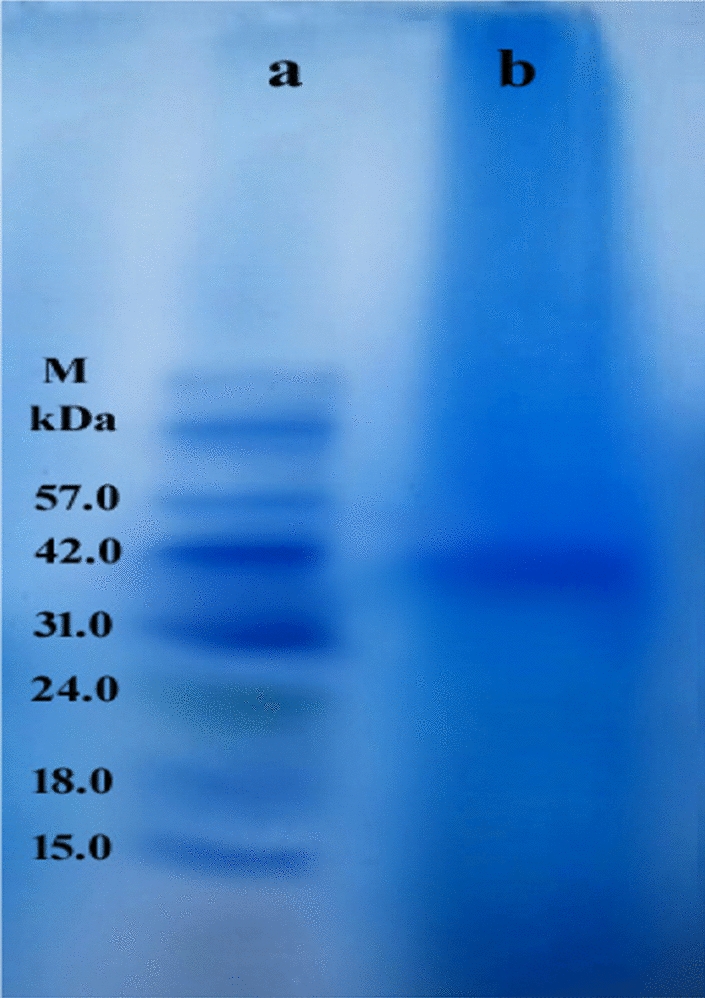
SDS–PAGE analysis with Lane **a** molecular weight markers, Lane **b** purified laccase from *Trichoderma harizanum* (PP389612)

The molecular weight and purity of laccase were determined using SDS-PAGE. As shown in Fig. 8**,** the standard protein marker and purified laccase are displayed in lanes (a) and (b), respectively. By using the SDS-PAGE method, the single protein band observed indicates that the laccase, purified from *Trichoderma harizanum* (PP389612), is highly purified. The relative molecular weight of purified laccase was found to be 41.00 kDa.

### Kinetics studies on laccase

**Fig. 9:**
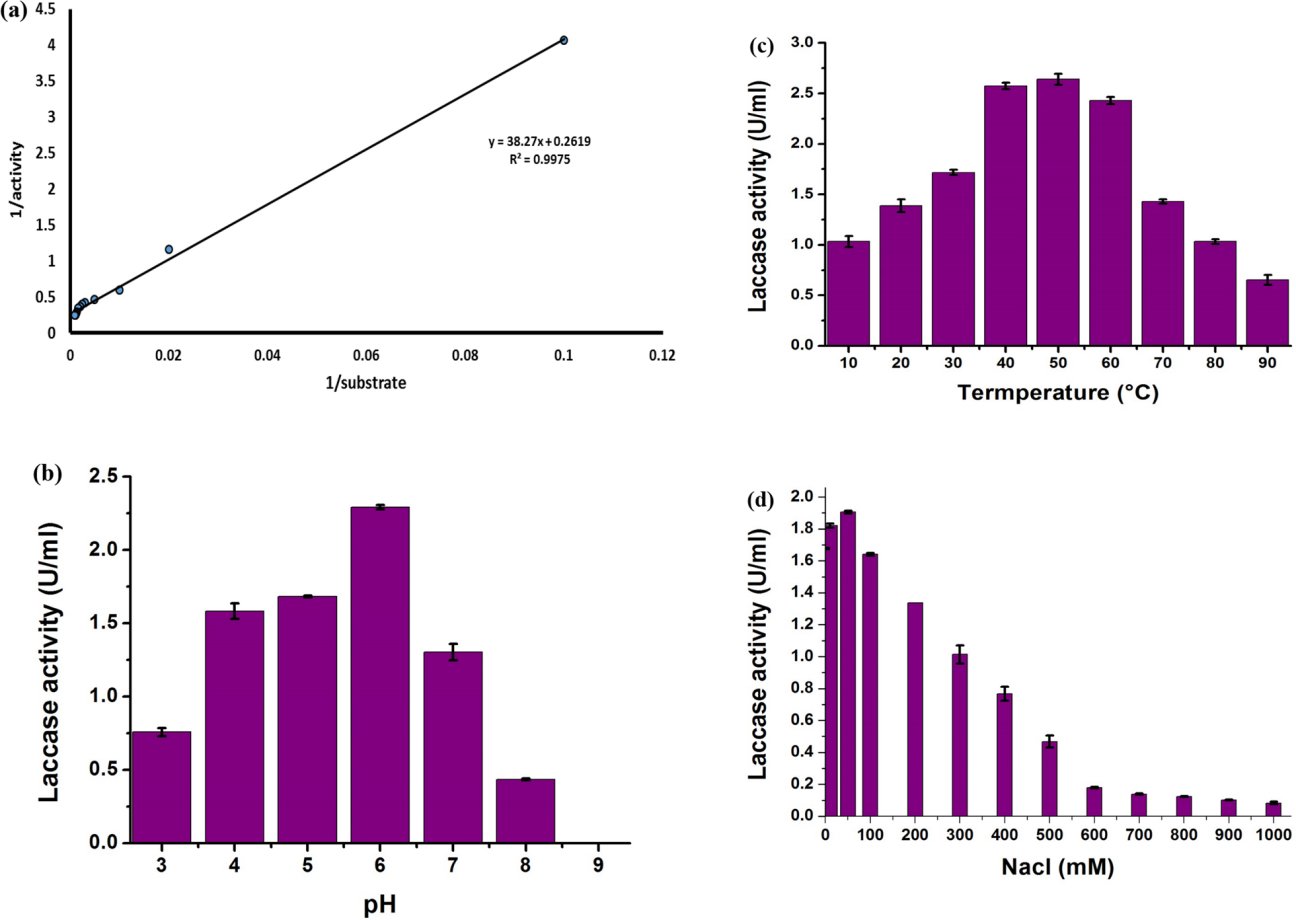
Kinetic studies of laccase activity from *Trichoderma harzianum* (PP389612) shows Lineweaver–Burk plot (**a**), pH dependency at 3, 4, 5, 6, 7, 8, and 9 pH (**b**), temperature dependency at 10, 20, 30, 40, 50, 60, 70, 80, and 90 °C (**c**), ionic strength using 10, 50, 100, 200, 300, 400, 500, 600, 700, 800, 900, and 1000 mM NaCl (**d**)

The kinetic properties of the precipitate enzyme, including K_m_, V_max_, pH, temperature, and ionic strength tolerance, were presented in Figs. 9 a-d. The Km values for *Trichoderma harzianum* (PP389612) Laccase were 146.12 μmol guaiacol and Vmax was 3.82 μmol guaiacol/ min Fig. 9a. Laccase activity was good in the pH 5–7 range, and the optimum pH for laccase activity was 6, giving 2.29 ± 0.015; however, the activity could not tolerate high alkaline pH and was stopped at pH 9 Fig. 9b. The enzyme tolerates a wide range of temperatures and has thermostability properties; the optimum temperature range for laccase activity was 40 to 50 ºC, giving 2.57 ± 0.03 and 2.64 ± 0.05 U/ml, respectively. The activity still works at high temperatures until 90 °C, giving 0.65 ± 0.05 U/ml, reflecting its tolerance, which could be considered a thermostable enzyme Fig. 9c. The optimum ionic strength for laccase activity was at 50 mM NaCl, giving 1.9 ± 0.008 U/ml, and the activity decreased after that until 0.083 ± 0.008 U/ml at 1000 mM NaCl, which reflects the high ionic strength tolerance of *Trichoderma harzianum* (PP389612) laccase Fig. 9d.

### Azo dyes degradation assay

**Fig. 10:**
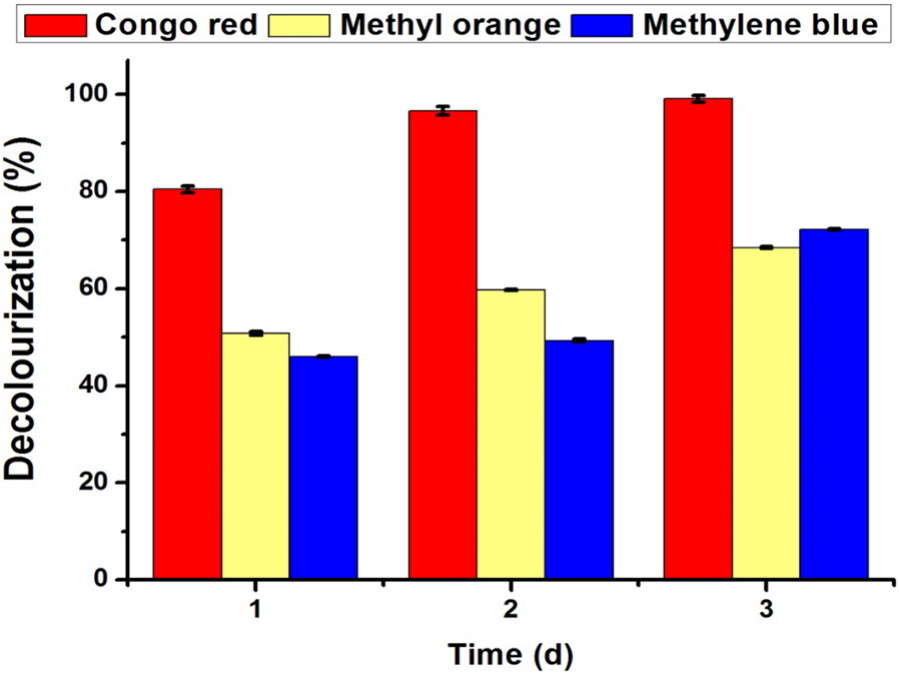
Decolourization efficiency of azo-dyes by *Trichoderma harzianum* (PP389612) laccase enzyme after 1 (24 h), 2 (48 h), and 3 (72 h) days at 28 °C for Congo red, methylene blue, and methyl orange

**Fig. 11:**
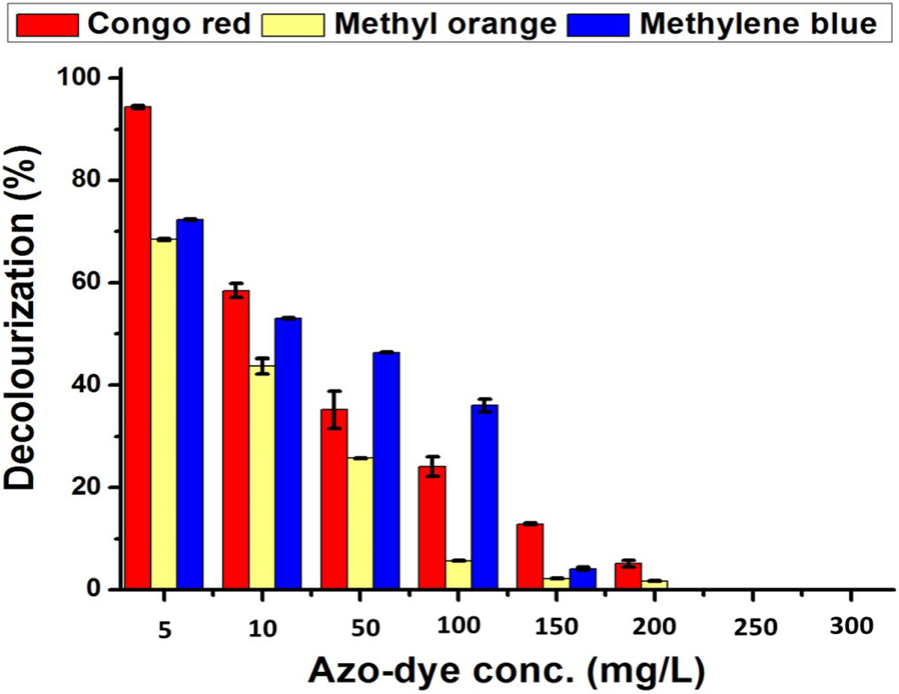
Decolourization efficiency of azo-dyes by *Trichoderma harzianum* (PP389612) laccase enzyme after three days of incubation at 28 °C using different concentrations of Congo red, methylene blue, and methyl orange (5–300 mg/L)

The decolorization efficiency of azo dyes by *Trichoderma harzianum* (PP389612) laccase after 24, 48, and 72 h for Congo red, methylene blue, and methyl orange is shown in Fig. 10. For all treatments, the decolorization efficiency of laccase increases with time and reaches its maximum after 72 h. The highest efficiency was achieved in Congo red decolorization, which reached 99% after 72 h., followed by methylene blue at 72% and methyl orange decolorization efficiency at 68.5%. As the concentration of azo dye escalates, there is a corresponding decline in decolorization efficiency. This trend continues until a point of inefficiency is reached at concentrations of 250 and 300 mg/L, as shown in Fig. 11. At 10 mg/L, the decolorization percentage was 58%, 44%, and 53%, while at 50 mg/L, it was 35%, 26%, and 46% for Congo red, methyl orange, and methylene blue, respectively.

## Discussion

Laccase oxidizes lignin by utilizing molecular oxygen and gives water as the only by-product, reflecting its safety. It also clears broad substrate specificity for aryl diamines, polyamines, phenols (ortho, para, amino, phenols, and polyphenols), and ascorbate [56]. Due to its low substrate specificity, laccase could be widely utilized as an environmental pollutant-degrading enzyme. Although laccase enzymes are found in nature, fungal laccases are more efficient for specific applications than laccases from other sources. The *Trichoderma* genus is recorded as multicultural, wide-spaced soil-borne fungus that could be found in various ecosystems [40]. This genus actively has a significant role in the natural biodegradation of lignocellulosic materials, which is attributed to this genus in agriculture waste management processes, bioremediation, and biotechnology fields. Moreover, *Trichoderma* is stated as a fungal source for industrial hydrolytic enzyme production [39].

Genus *Trichoderma* is known for its high enzyme production abilities, particularly the laccase. In their species, *T. atroviride* and *T. harzianum* were recorded as the most vital laccase producers compared with other *Trichoderma* species [16, 57]. Singh et al. [58] and Nuhu et al. [59] revealed that laccase production from *Trichoderma* sp. was affected highly by the incubation period and the temperature and found that incubation for 6–9 days at 25–30 °C was convenient for laccase production by *Trichoderma* species. In this study, nine isolates of *Trichoderma* (four isolates of each *T. atroviride* and *T. harzianum* and one isolate of *T. koningi*) were collected from different sources, identified, and screened for laccase secretion. Eight of them were recorded as laccase producers. *Trichoderma harzianum* (PP389612) was the superior, giving 1.6 ± 0.045 U/ml, followed by *T. atroviride* ASU 333, which yielded 1.43 ± 0.079 U/ml after 6 days of incubation at 28 °C. In agreement with our findings, Ranimol er al. [60] obtained 0.971 U/ml laccase from *Trichoderma harzianum* using a mineral medium containing 1% glucose, 0.05% guaiacol, and pH 7 at 30 °C. Divya et al. [61] obtained 0.678 U/mL laccase from *Trichoderma viride* after 96 h incubation at 30 °C using a mineral medium containing 1% glucose and an initial pH of 6.0. Umar [43] evaluated ten *Trichoderma* species for laccase production and found that *T. atroviride* was the highest producer with 2.62 U/ml. Mohsen et al. [62] obtained 0.266 U/ml laccase from *Trichoderma viride* using 0.02% guaiacol after 96-h incubation at 28 °C. Abd El Monssef et al. [63] reported that *Trichoderma harzianum* produced 1.286 U/ml after 6 days of incubation at 30 °C using a medium supplemented by 0.04% guaiacol and an initial pH of 5.5.

On the other hand, the isolate of *T. koningii* tested in this study did not produce any detectable amount of laccase. This finding is consistent with the results reported by Ahmed and Siddiqui [64]. In their study, they examined 29 different isolates from seven Trichoderma species for laccase secretion and found that *T. harzianum* and *T. viridae* were laccase producers, whereas *T. koningii, T. gamsii, T. hermatum*, and *T. aeroviridae* produced very low or negligible amounts. Advances in fungal molecular biology, especially in DNA analysis, improve taxonomy, justifying multiphasic approximations in studies of different fungal groups.

Furthermore, the fungal phylogenetic concept of species considers the evolutionary and genealogical connections between organisms in each group. A *Trichoderma harzianum* isolate was identified at the molecular level after morphological description using ITS 1 and ITS 4 sequences that were amplified for about 586 base pairs. Researchers Menezes et al. [65] and Hermosa et al. [66] used molecular methods to identify different types of *Trichoderma* spp., including *T. harzianum.* When they amplified ITS regions, they got 560–600 bp bands.

In general, laccase production by native fungi could be observed in low concentrations; however, its concentration could rise quickly through optimized cultural conditions. Also, microbial enzymes with various desired characteristics can be obtained by optimizing the production conditions [67]. According to Kumar and Takagi [68] and Niyonzima et al. [67], no common growth medium could provide the optimum industrial production of enzymes by fungi or bacteria; each microbial species has its own optimum growth and production conditions. However, Hajji et al. [69] revealed that statistical optimization methods were more efficient than one parameter at a time method for microbial industrial enzyme production. The Plackett–Burman design was used to look at the nutritional and growing factors and pick out the most important and useful ones for laccase production by *T. harzianum* PP389612 (the best producer)**.** The significant variables that greatly affected the laccase production process were incubation temperature, time, peptone, KH_2_PO_4_, K_2_HPO_4,_ and guaiacol concentration.

The incubation temperature and time are vital environmental factors that control industrial enzyme production by microorganisms by controlling the growth pattern of fungi and, thus, enzyme production by the microbe [67]. It was stated that laccase production from Trichoderma sp. was obtained with high concentrations after 6–9 days of incubation at 25–30 °C Singh et al. [58] and Nuhu et al. [59]. Also, nitrogen sources play an essential role in microbial cell walls, proteins involving enzymes, amino acids, peptides, and nucleotide synthesis [70]. Organic nitrogen sources like peptone and yeast extract give the highest industrial enzyme production, as stated by Shwetha et al. [71] and Nuhu et al. [59].

Minerals and metal ions significantly impact on the laccase production process because they control the initial stages of the catalytic reaction. However, excessive concentrations of these compounds slow production and even inhibit the whole process [72]. The highest laccase production was 2.89 U/ml (predicted value of 2.71 U/ml) obtained in the run (6) with shaking fermentation at 25 °C and pH 7 after 8 days using a 2% inoculum size of 50 ml fermentation medium involving 1.5% glucose, 0.6% peptone, 0.1% KH_2_PO_4_, 0.01% K_2_HPO_4,_ and 50 μg/l guaiacol. After optimization, the laccase activity recorded in our study was higher than those recorded by Mohsen et al. [62], who obtained 1.03 U/ml laccase from *Trichoderma viride* after 96 h of incubation at 30 °C, Umar [43] found that the highest laccase activity from *T. atroviride* was 2.62 U/ml. After the laccase optimization process, Jenefar et al. [73] obtained 2.3 U/ml laccase from *Perenniporia subtephropora* using medium initial pH 6 and incubation at 30 °C, while Sadhasivam et al. [74] utilized *Trichoderma harzianum* by augmented 1 mM CuSO_4_ and found that the highest yield of laccase activity reached 4.36 U/ml after 4 days. Moreover, Bagewadi et al. [17] used the Plackett–Burman design to select the factors significantly affecting laccase enzyme production by *T. harzianum* HZN10 under solid-state fermentation, They found that glucose, yeast extract, wheat bran, and Cu^2+^ ions are key fermentation variables. Sabarathinam et al. [75] found that the maximal yield of laccase by an isolate of *T. asperellum* was obtained in the medium having the composition of 1% yeast extract, 1% glucose, 0.02% ammonium tartrate, 0.5 mM inducer, 15% mineral salt solution, and 5% inoculum at pH 4.5.

In our findings during the study, laccase purified from *Trichoderma harizanum* (PP389612) was shown as a single protein band with a 41.00 kDa molecular weight in SDS-PAGE, which was in agreement with More et al. [76], and Ali et al. [77]. Bagewadi [17] purified 56.0 kDa laccase from *Trichoderma harzianum* HZN10. Meanwhile, two laccase isozymes from *Trichoderma harzianum* S7113 were isolated at molecular weights of 48 and 63 kDa by Elsayed et al. [78]. These variations were noted in the molecular sizes of the laccase enzyme generated from the same organism, *T. harizanum* isolates. these could be attributed to changes throughout the glycosylation patterns that appear with structural and chemical changes of the post-immobilization of protein [79]. Moreover, another agreement was obtained by Giardina et al. [80], which suggests that the laccase enzyme could act as a monomer enzyme.

Some of the kinetic properties of the precipitate laccase produced by *T. harzianum* (PP389612), such as K_m_, V_max_, pH, temperature, and ionic strength tolerance, were examined**.** From the enzyme’s kinetic parameters, K m and V max are indicators of enzyme–substrate specificity, where lower Km indicates high binding abilities between the enzyme and the substrate and high substrate specificity [81]. In our study, the K_m_ value was 146.12 μmol guaiacol, while the V_max_ was 3.82 μmol guaiacol/min. It was found that K_m_ and V_max_ values are sensitive to microbial growth conditions like inhibitors or activators presence, temperature, and pH, which can change the K_m_ and V_max_ from one study to another and the enzyme behavior [82]. Zhuo et al. [55] reported that K_m_ and V_max_ of purified laccase from *Pleurotus ostreatus* were 663.021 μmol guaiacol and 0.048 μmol guaiacol/min, while Sharma et al. [83] found that Km was 217 μM guaiacol and Vmax of 92.59 μmol guaiacol/min of laccase extracted from *Ganoderma* sp. However, laccase extracted from *Chaetomium thermophilium* has a K_m_ value of 400 μmol guaiacol and a V_max_ of 2.5 μmol guaiacol/min [45].

pH represents one of the most critical parameters for enzymatic performance. The pH can influence the whole enzymatic industrial process as the pH value could alter the shape and even the size of the enzyme used, which could affect the enzymatic specificity and its affinity to combine with the substrate [84]. It was found that fungal laccase is active at low pH values from 3 to 6 and loses its activity at high alkalinity [85–87], which was in the same line with our findings. In our study, laccase activity was stable in the pH range of 5–7, and the maximum pH was 6, giving 2.29 ± 0.015 U/ml. However, the laccase activity could not tolerate a high alkaline pH and was stopped at pH 9. Ranimol et al. [60] stated that laccase extracted from *Trichoderma harzianum* has maximum activity at pH 5.5 by 0.681 U/ml. Chefetz [45] found that the optimum pH for laccase activity produced by *Chaetomium thermophilium* was 6, while EI-Fakharany et al. [88] observed that at pH 5, laccase enzyme extracted from *Fusarium oxysporum* has high activity. Ahmed and Siddiqui [64] examined the influence of pH on the stability of laccase and recorded that it was stable at pH 4.5. However, as pH became alkaline or acidic, the stability of the laccase decreased. However, Chefetz [45] and Zhuo et al. [55] found that the laccase enzyme was stable at pH ranges of 5 to 10, and laccase enzyme could keep about 90% of its activity at pH ranges of 7 to 10 within 24 h.

Enzymatic heat stability is critical for enhancing and developing industrial operations; thermostable enzymes minimize microbial contamination risks, reduce substrate viscosity, and increase solubility, enhancing the process speed [89, 90]. Also, thermostable enzymes usually resist proteolysis and denaturation substances, which are essential characteristics for commercial preparations and environmental applications [89, 91]. Thermostable enzymes can be identified as enzymes that can withstand 50 °C without losing their activity or altering their structure [90]. The tested laccase tolerates a wide range of temperatures and has thermostability properties, with an optimum temperature range of 40 to 50 °C, giving 2.57 to 2.64 U/ml. The activity still works at high temperatures until 90゜C, giving 0.65 ± 0.05 U/ml, reflecting its thermos tolerance. Nearly similar results were reported by several studies. Nishizawa [86], Munoz [87], and Youn et al. [92] found that the optimal temperature range for fungal laccase activity was 30–60 °C. Elsayed et al. [78] reported that two *T. harzianum* S7113 laccase isoenzymes gave their optimum activity at 50 °C and then started to decrease. Chefetz [45] reported that the laccase produced by *Chaetomium thermophilium* had a temperature optimum between 50 and 60 °C, was stable for 1 h at 70 °C, and had half-lives of 24 and 12 h at 40 and 50 °C, respectively. Xu et al. [93] reported that a *Tricholoma matsutake* laccase has high thermal stability properties and maintains most of its activity between temperature ranges from 20 °C to 80 °C. On the other hand, Ahmed and Siddiqui [64] found that the relative activity of laccase produced by *T. harzianum* had a minor effect at temperatures ranging from 20 to 40 °C; however, at temperatures ranging from 50 to 70 °C, the enzyme became inactive as the time of storage increased.

Enzymatic ionic strength influences the enzymatic activities, the kinetics of the hydrolysis, and even the composition of the final products [94]. The optimum ionic strength for laccase activity was at 50 mM NaCl, giving 1.9 ± 0.008 U/ml, and the activity decreased after that until 0.083 ± 0.008 U/ml at 1000 mM NaCl, which reflects the high ionic strength tolerance of *Trichoderma harzianum* (PP389612) laccase. Elsayed et al. [78] found that adding sodium salt decreased laccase isozyme B activity from *T. harzianum*, while isozyme laccase A activity increased. Mukhopadhyay and Banerjee [95] found that 1 mM sodium salt decreased laccase activity in *Trametes versicolor*. Chairin et al. [96] recorded that sodium chloride has an increasing inhibitory effect with increasing its concentration on *Trametes polyzona* and *T. versicolor* laccase and a reversible effect of more than 50% at 20 mM NaCl.

Laccase enzymes are a valuable natural agent for azo dye degradation using their oxidative properties [97]. It has unique capabilities that can be utilized in the bioremediation of water and soil pollution that is elaborated on in the environment through different industries [56]. Therefore, in our study, the decolorization efficiency of azo dyes by tested laccase after 24, 48, and 72 h for Congo red, methylene blue, and methyl orange was studied**.** The decolorization efficiency was increased by increasing the time and reached its maximum after 72 h. The highest efficiency was achieved in Congo red decolorization, which reached 99% after 72 h, followed by methylene blue at 72% and methyl orange decolorization at 68.5%. Increasing the azo-dye concentrations decreases the decolorization efficiency until the loss efficiency reaches 250 and 300 mg/L. Laccase enzyme was utilized to decolorize the malachite green dye, which aquaculture fish farms use to control harmful protozoan and fungal infections [98].

## Conclusion

The use of enzymes in bioremediation has received extraordinary attention in both academic and industrial sectors instead of living microbial cells, as living cells need nutrients and produce unwanted metabolic compounds that require expensive economic purification. In addition, enzymes are highly specialized in performing the required reaction. In this study by *Trichoderma harzianum* (PP389612), laccase production activity was 2.89 U/ml. The purified laccase appeared as a single protein band with a molecular weight of 41.00 kDa in SDS-PAGE, with K_m_ and V_max_ values of 146.12 μmol guaiacol and 3.82 μmol guaiacol/min. This enzyme was active and stable in a wide pH range and had thermostability properties with an optimum temperature range of 40 to 50 °C. It has a high ionic strength against sodium chloride. It is worth mentioning that the extracted laccase is highly effective in removing of azo dyes. The highest laccase decolorization efficiency was reached at 99%, 72%, and 68.5% after 72 h for Congo red, methylene blue, and methyl orange, respectively.

## Data Availability

The authors confirm that the data supporting the findings of this study are available within the article and indicated supplementary materials. Sequence data that support the findings of this study have been deposited in the NCBI with the accession code *Trichoderma harzianum* (Accession no. PP389612) (https://www.ncbi.nlm.nih.gov/nuccore/PP389612).
